# Canine dental pulp stem cell-derived exosomes: Proteomic characterization and first xenogeneic application for post-castration wound healing in cats

**DOI:** 10.14202/vetworld.2026.3755-3773

**Published:** 2026-08-27

**Authors:** Yustina Septi Dyanitha, Aris Haryanto, Teguh Budipitojo, Dito Anggoro, Chenphop Sawangmake, Sirirat Rattanapuchpong, Medania Purwaningrum

**Affiliations:** 1 Postgraduate Student, Faculty of Veterinary Medicine, Universitas Gadjah Mada, Yogyakarta, Indonesia.; 2 Department of Biochemistry and Molecular Biology, Faculty of Veterinary Medicine, Universitas Gadjah Mada, Yogyakarta, Indonesia.; 3 Department of Anatomy, Faculty of Veterinary Medicine, Universitas Gadjah Mada, Yogyakarta, Indonesia.; 4 Department of Surgery and Radiology, Faculty of Veterinary Medicine, Universitas Gadjah Mada, Yogyakarta, Indonesia.; 5 Veterinary Stem Cell and Bioengineering Innovation Center, Veterinary Pharmacology and Stem Cell Research Laboratory, Faculty of Veterinary Science, Chulalongkorn University, Bangkok, Thailand.; 6 Veterinary Stem Cell and Bioengineering Innovation Center, Faculty of Veterinary Science, Chulalongkorn University, Bangkok, Thailand.; 7 Academic Affairs, Faculty of Veterinary Science, Chulalongkorn University, Bangkok, Thailand.

**Keywords:** angiogenesis, cell-free therapy, dental pulp stem cells, exosomes, proteomics, regenerative medicine, wound-healing, xenogeneic therapy

## Abstract

**Background and Aim:**

Mesenchymal stem cell-derived exosomes (MSC-Exos) have emerged as promising cell-free therapeutics because they retain the regenerative and immunomodulatory properties of their parent cells while minimizing the risks associated with cell transplantation. Canine dental pulp stem cell-derived exosomes (cDPSC-Exos) represent an attractive veterinary regenerative platform because dental pulp is readily accessible through minimally invasive procedures. However, their molecular composition and xenogeneic therapeutic potential remain largely unexplored. This study aimed to characterize canine dental pulp stem cells (cDPSCs) and their derived exosomes, define the proteomic profile of cDPSC-Exos, and evaluate their safety and therapeutic potential for post-castration wound-healing in cats.

**Materials and Methods:**

cDPSCs were isolated from canine dental pulp and characterized according to mesenchymal stem cell (MSC) criteria using morphology, colony-forming ability, proliferation, trilineage differentiation, and reverse transcription-quantitative polymerase chain reaction analysis of MSC surface and stemness markers. Exosomes were isolated from conditioned medium and characterized by nanoparticle tracking analysis and *CD9* expression. High-resolution mass spectrometry was used for proteomic profiling, followed by functional pathway and protein interaction analyses. A randomized controlled pilot study involving six healthy male cats (n = 3/group) compared topical cDPSC-Exos gel (3.3 × 10⁸ particles/g) with Bioplacenton® following castration. Wound-healing was evaluated using macroscopic wound scores, hematological parameters, and laser speckle contrast imaging.

**Results:**

The isolated cDPSCs fulfilled the defining characteristics of MSCs, including multilineage differentiation and expression of MSC-associated markers. cDPSC-Exos exhibited typical exosome characteristics, with a mean particle diameter of 111 ± 24 nm. Proteomic analysis identified 312 proteins, including 22 proteins associated with inflammation regulation, angiogenesis, extracellular matrix organization, cell migration, and tissue remodeling. Western blot analysis confirmed the presence of vimentin and β-actin, supporting the proteomic findings. Topical administration of cDPSC-Exos was well tolerated, without adverse reactions, and significantly improved wound redness, swelling, and pain scores during early healing (p < 0.05), while hematological variables and tissue perfusion remained comparable to the positive control.

**Conclusion:**

This study provides the first comprehensive proteomic characterization of cDPSC-Exos and the first evidence supporting their xenogeneic application in feline post-castration wound-healing. The findings demonstrate that cDPSC-Exos constitute a safe and promising cell-free regenerative therapy capable of modulating early wound repair through multiple biological pathways. Larger controlled studies are warranted to validate their clinical efficacy and optimize therapeutic protocols.

## INTRODUCTION

Mesenchymal stem cell (MSC)-based regenerative therapy has demonstrated therapeutic potential for various diseases, including cancer, heart failure, stroke, neurological disorders, diabetes mellitus, autoimmune diseases, and chronic inflammation, owing to their regenerative capacity, immunomodulatory effects, and secretion of bioactive factors that promote tissue repair and modulate inflammation [1–5]. Furthermore, MSCs can be isolated from various tissue sources, including bone marrow, adipose tissue, placenta, umbilical cord, hair follicles, and dental tissue, across various animal species, thereby supporting the development and large-scale production of MSCs [6–10]. The therapeutic effects of MSCs are partly mediated by paracrine mechanisms through the secretion of extracellular vesicles (EVs), particularly exosomes, which play a crucial role in cellular communication, supporting cellular functions [11, 12].

Exosomes are a nanoscale subpopulation of EVs with diameters ranging from 30 to 150 nm that contain bioactive molecules, including nucleic acids, lipids, and proteins, to support cellular functions [12, 13]. The biological properties of exosomes make them suitable as cell-free therapy agents, with clinical potential comparable to cell-based therapy and a lower risk of immune rejection, tumorigenesis, toxicity, and ethical concerns. This enables scalable storage and distribution, as exosomes do not contain viable cells and exhibit lower expression of immunogenic surface molecules [12, 14]. The lower biosafety risks associated with exosome-based therapy also enable xenogeneic administration across species [12, 15, 16]. Previous studies have reported the safety and therapeutic efficacy of human MSC-derived exosomes in canine, feline, and laboratory animal models, suggesting a relatively conserved mechanism of exosome-mediated tissue repair across species barriers [15, 17–21].

Several preclinical and clinical studies have demonstrated the safety and therapeutic efficacy of cell-free therapy using MSC-derived exosomes in the treatment of neurodegenerative diseases, periodontal tissue regeneration, osteoarthritis, and other degenerative disorders [22–27]. Previous studies also suggest that MSC-Exos accelerate wound-healing and enhance tissue repair in animal models, with human dental pulp stem cell-derived exosomes (huDPSC-Exos) shown to support angiogenesis, inflammatory responses, and immunomodulation during the wound-healing process [6, 11, 13, 28]. These regenerative properties highlight the potential application of MSC-Exos for postoperative wound-healing in animals, which remain susceptible to post-surgical complications such as wound dehiscence, prolonged inflammation, infection, and septicemia, thereby delaying recovery and compromising animal welfare [29–32].

Canine dental pulp stem cells (*cDPSCs*) are promising candidates for regenerative therapy in veterinary medicine because of the minimally invasive, ethically favorable dental pulp collection, and various studies have reported their MSC-like characteristics based on the minimum criteria established by the International Society for Cellular Therapy (ISCT) [33, 34]. However, their exosomes (*cDPSC-Exos*) remain largely unexplored, and no studies have evaluated the xenogeneic potential of *cDPSC-Exos* in cats. Comprehensive proteomic characterization of *cDPSC-Exos* is also lacking.

The objective of this pilot study was to characterize *cDPSCs* and *cDPSC-Exos* and to evaluate their therapeutic potential through proteomic profiling and *in vivo* xenogeneic application for postoperative wound-healing. This is the first study to combine *cDPSC* characterization, *cDPSC-Exos* characterization and proteomic profiling, with xenogeneic evaluation in cats post-castration wounds as a standardized, clinically relevant, and naturally occurring surgical model. We hypothesized that *cDPSC-Exos* would promote postoperative wound-healing in cats through regulation of inflammation, immunomodulation, and angiogenesis.

## MATERIALS AND METHODS:

### Ethical approval 

This study was conducted in accordance with accepted animal welfare principles and the ARRIVE 2.0 guidelines. The experimental protocol was reviewed and approved by the Ethics Committee, Faculty of Veterinary Medicine, Universitas Gadjah Mada, Indonesia, under approval number 101/EC-FKH/Int./2025. Canine dental pulp tissue was obtained from a healthy donor dog during routine dental extraction with appropriate clinical oversight. The feline clinical evaluation was conducted using client-owned healthy male cats undergoing elective castration at Prof. Soeparwi Animal Hospital, Faculty of Veterinary Medicine, Universitas Gadjah Mada, after obtaining written informed consent from the owners.

All cats received standardized perioperative care, including anesthesia, analgesia, antibiotic coverage, postoperative monitoring, and daily wound assessment. Animals were housed individually with free access to food and water, and all procedures were performed by qualified veterinary personnel to minimize pain, stress, and discomfort. The study used a positive-control design because leaving post-surgical wounds untreated was considered ethically inappropriate. No adverse reactions, wound dehiscence, infection, or clinically relevant systemic complications were observed during the study period.

### Study period and location 

This study was conducted from January 2025 to January 2026 at the Integrated Laboratory, Faculty of Veterinary Medicine, Gadjah Mada University for cell isolation and culture; the Laboratory of Biochemistry and Molecular Biology, Faculty of Veterinary Medicine, Universitas Gadjah Mada for *cDPSCs* and *cDPSC-Exos* characterization; and Prof. Soeparwi Animal Hospital, Faculty of Veterinary Medicine, Universitas Gadjah Mada for *in vivo* observation.

### Study design 

**Figure 1:**
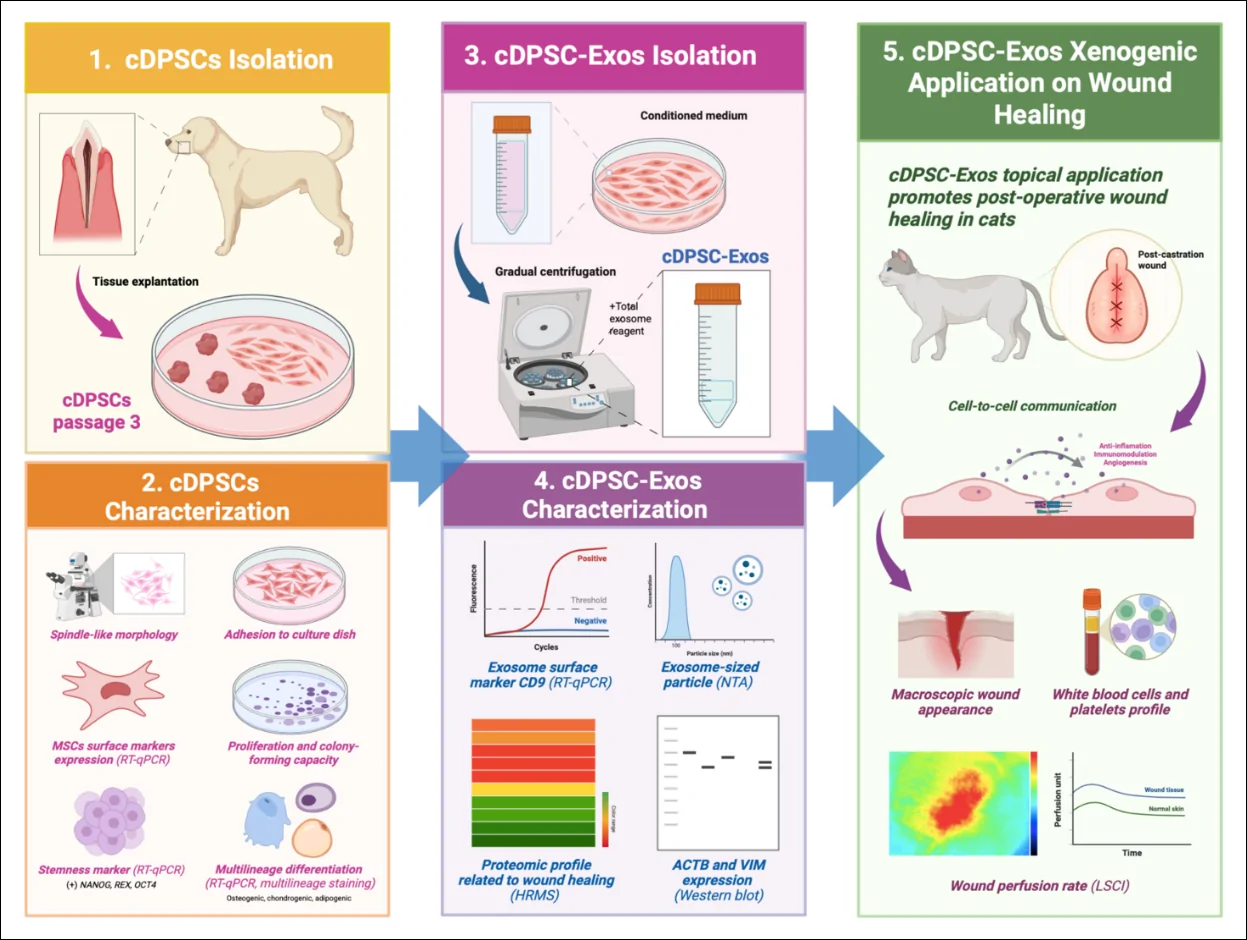
Schematic overview of canine dental pulp stem cells (cDPSCs) isolation and characterization; cDPSC-derived exosome (cDPSC-Exos) isolation, characterization, and proteomic profiling; followed by xenogeneic topical application of cDPSC-Exos to promote postoperative wound-healing in cats through cell-to-cell communication, modulation of immune responses, reduction of inflammation, and stimulation of angiogenesis.

This study employed a combined *in vitro* and in vivo approach consisting of cDPSC isolation and characterization; cDPSC-Exos isolation, characterization, and proteomic profiling; and a randomized controlled pilot study for xenogeneic evaluation of post-castration wound-healing in cats, as demonstrated in Figure 1.

### Cell isolation, culture, and expansion

Dental pulp was obtained from a single healthy permanent molar of a 2.5-year-old domestic male dog with no history of systemic disease or oral pathology. Isolation involving tissue explant technique was adapted from previous work [7, 35]. The crown of the molar tooth was sectioned to collect the dental pulp. The dental pulp was minced to ±0.5 cm and placed into a 35 mm culture plate containing Dulbecco’s modified Eagle medium (Gibco™, Thermo Fisher Scientific, Waltham, MA, USA) medium supplemented with 10% fetal bovine serum (Gibco™), 1% antibiotic-antimycotic (Gibco™), and 1% GlutaMAX (Gibco™); maintained in 70% humidity; and incubated at 37°C in 5% CO₂ with medium replacement every 48 h. When the cells reached approximately 80% confluence, they were subcultured into three new 35 mm culture plates containing medium. Passage 3 of the *cDPSCs* was used for characterization based on MSCs’ minimal requirements by ISCT.

### Colony-forming assay

Isolated *cDPSCs* were cultured at a concentration of 500 cells per 60 mm culture dish (TPP®, Trasadingen, Switzerland) for 14 days, following the previously published protocol [7, 36]. After 14 days, colonies were washed with Phosphate-buffered saline (PBS, pH 7.4), fixed with cold methanol for 20 min, stained with crystal violet for 5 min, and colonies containing ≥50 cells were counted [7, 35]. Colony-forming efficiency (CFE) was calculated using the formula CFE(%) = (number of colonies formed/number of cells seeded) × 100%.

### Proliferation assay

*cDPSCs* were incubated in culture medium with 5% alamarBlue™ (Sigma-Aldrich, St. Louis, MO, USA) for 3 hours to assess the proliferation capability on days 1, 5, and 7 [7, 35]. The absorbance was recorded at 570 nm, and cell viability was quantified as the percentage reduction of alamarBlue according to the product’s guidelines.

### Multilineage differentiation

The *cDPSCs* multilineage differentiation, including adipogenesis, osteogenesis, and chondrogenesis, was assessed *in vitro* using a previously published protocol [7, 10, 13, 37, 38]. *cDPSCs* were seeded into three separate 24-well plates and cultured in osteogenic, chondrogenic, or adipogenic induction media formulated as previously described [7, 38, 39], followed by Alizarin Red (Sigma-Aldrich) staining to detect calcium deposition in osteogenic differentiation, Alcian Blue (Sigma-Aldrich) staining to detect glycosaminoglycan (GAG) accumulation in chondrogenic differentiation, and Oil Red O (Sigma-Aldrich) staining to assess adipogenic differentiation based on intracellular lipid droplet formation.

### Reverse transcription-quantitative polymerase chain reaction (RT-qPCR)

The gene expression of MSC surface markers (*CD90*, *CD44*, *CD105*, *CD73*, and *CD45*) was assessed using RT-qPCR as an alternative approach to flow cytometry. RT-qPCR was also used to assess stemness-related markers (*NANOG*, *REX-1*, *OCT-4*), osteogenic differentiation markers (*RUNX-2*, *OSX*, *OCN*), chondrogenic differentiation markers (*COL-X*, *SOX-9*), and adipogenic differentiation markers (*PPAR-γ*, *LPL*) of *cDPSCs*. Specific primers used in this study are presented in Table 1.

**Table 1:** List of primer sequences used to characterize cDPSCs and cDPSC-Exos.

**Gene**	**Forward primer sequence (5’-3’)**	**Reverse primer sequence (5’-3’)**
GAPDH	CCAACTGCTTGGCTCCTCTA	GTCTTCTGGGTGGCAGTGAT
CD90	AGGACGAGGGGACATACACA	ATGCCCTCACACTTGACCAG
CD44	CCCCATTACCAAAGACCACGA	TGGGATTTGAGGTTTCCGCA
CD105	CGAGGAGTCTGTCACCGGAAA	GCGCCAAAGGTGATACCCAG
CD73	GGCAACCTGATTTGTGATGCT	AGGTAATTGTGCCGTTGTTCC
CD45	GTTTCCAGTTCTGTTTCCCCAG	CATTGGTCACAATTCACGGTATCA
NANOG	CAGCAGATGCAAGAACTTTCCA	AGCAGGTACCCCTGAGTCAC
REX-1	AGGTTCTCACAGCAAGCTCA	CCAGCAAATTCTGCGCACTG
OCT-4	AGGAGAAGCTGGAGCAAAACC	GTGATCCTCTTCTGCTTCAGGA
COL-X	AGCACCCCGAATCCATCTGAG	TGCCCGTAGGTGTTTGGTATC
SOX-9	TCTGGAGGCTGCTGAACGA	TTCTTCACCGACTTCCTCCG
RUNX-2	GGAAGAGGCAAGAGTTTCACC	GTGCTCACTTGCCAACAGAA
OSX	GCGTCCTCCCTGCTTGAG	GCTTTGCCCAGTGTCGTTG
OCN	GCCAGCCTATGGTCTCCTCTG	CCACCAGCTCCTTCTGTTCTCT
PPAR-γ	CCTCTTCCATGCTGTTATGGGT	TGGCATCTCTGTGTCAACCA
LPL	CTGGAGAGACTCAGAAAAAGGTAAT	TCCTTCTGTAGATTTGCTCAGGT
CD9	ATTTCGTCTTCTGGCTTGCTGG	AGGGCACCAGCTCCAATCAG

Total RNA was extracted using TRIzol® reagent (Invitrogen, Carlsbad, CA, USA) and Direct-zol™ RNA Miniprep kit (Zymo Research, Irvine, CA, USA); with RNA quantification performed using NanoDrop™ (Thermo Fisher Scientific, Waltham, MA, USA). Reverse transcription was performed using QuantiNova® Reverse Transcription kit (Qiagen, Hilden, Germany), followed by qPCR amplification using QuantiNova® SYBR® Green PCR kit (Qiagen) on Rotor-Gene Q real-time PCR cycler (Qiagen). The mRNA expression was normalized to the housekeeping gene (*GAPDH*) using the formula ΔCt = Ct target gene - Ct GAPDH for stemness and differentiation markers, while surface marker expression was presented as a percentage calculated using the formula 2^−ΔCt^ × 100% [7, 35, 40].

### Exosome isolation and characterization 

Exosome isolation was performed using Total Exosome Isolation Reagent (Invitrogen, catalog no. 4478359) following the product’s protocol as previously reported in MSC-derived exosome studies [41, 42]. Conditioned medium (CM) from passage 3 *cDPSC* cultures was collected and centrifuged at 2,000 × *g* for 30 min at 4 °C to obtain a cell- and debris-free supernatant. Total Exosome Isolation Reagent (Invitrogen) was added to the conditioned medium at 25% of the conditioned medium volume, and the mixture was incubated overnight at 4 °C. Following incubation, centrifugation at 10,000 × *g* for 60 min at 4 °C was performed to collect the exosomal pellet, which was resuspended in 5 mL of sterile PBS and stored at −80 °C until further use. Characterization of *cDPSC-Exos* was performed by evaluating *CD9* transcript expression using RT-qPCR, while particle size distribution was assessed using nanoparticle tracking analysis (NTA).

### Exosomes proteomic profiling 

Proteomic analysis of *cDPSC-Exos* was performed using an Orbitrap Exploris 240 high-resolution mass spectrometer (Thermo Fisher Scientific) following previously reported MSC-derived exosome proteomic workflows [43, 44]. Samples were lysed using Sodium dodecyl sulfate-based lysis buffer, reduced with dithiothreitol, digested with MS-grade trypsin, acidified with 1% trifluoroacetic acid (TFA), and filtered prior to analysis. Peptide separation was carried out using a Vanquish Horizon Ultra-high-performance liquid chromatography system equipped with an Acclaim PepMap 100 C18 column, followed by analysis on an Orbitrap Exploris 240 high-resolution mass spectrometer (Thermo Fisher Scientific) operating in positive ion mode with Full MS/dd-MS² acquisition. Raw data were processed using Proteome Discoverer 2.5 with the SequestHT search engine against the UniProt (*Canis lupus familiaris*) database. Functional enrichment analysis was performed using Reactome, and protein-protein interaction (PPI) networks were analyzed using STRING. Due to the limited annotation available in the *C. l.* *familiaris* database, functional and pathway analyses related to wound-healing were conducted using the *Homo sapiens* database to improve biological interpretation.

### Western blot analysis 

Western blot analysis was performed to validate the proteomic findings by assessing vimentin expression using the iBlot™ 2 and iBind™ Western Systems (Thermo Fisher Scientific) following a previously reported workflow [45]. Protein concentrations were determined using a BCA Protein Assay Kit (Thermo Fisher Scientific), separated using Sodium dodecyl sulfate-polyacrylamide gel electrophoresis on 4-12% Bis-Tris gels, and transferred onto polyvinylidene difluoride (PVDF) membranes using the iBlot™ 2 Transfer System (Thermo Fisher Scientific). Membranes were then blocked and incubated with mouse anti-vimentin monoclonal antibody (V9) and rabbit anti-β-actin antibody as the internal loading control (Thermo Fisher Scientific), followed by Horseradish peroxidase-conjugated rabbit anti-mouse immunoglobulin (Ig)G and goat anti-rabbit IgG secondary antibodies (Thermo Fisher Scientific). Protein bands were visualized using SuperSignal™ West Pico PLUS Chemiluminescent Substrate (Thermo Fisher Scientific) according to the manufacturer’s instructions.

### Xenogeneic clinical evaluation of postoperative wound-healing in cats 

Clinical evaluation of xenogeneic *cDPSC-Exos* therapy was conducted in six clinically healthy male cats aged ≤5 years, which were randomly assigned by simple randomization into two groups: (1) the *cDPSC-Exos* group, receiving topical *cDPSC-Exos* gel on post-castration wounds, and (2) the positive control group, receiving topical Bioplacenton®. The absence of a negative control group was based on ethical considerations regarding animal welfare, since leaving post-surgical wounds untreated could potentially lead to adverse complications.

All cats underwent castration at Prof. Soeparwi Animal Hospital, Faculty of Veterinary Medicine, Gadjah Mada University, following owner-informed consent. Cats were housed individually with *ad libitum* access to food and water and received the same anesthesia, analgesia, antibiotic, and postoperative care protocols. Tramadol (2-4 mg/kg IV), ranitidine (1-2 mg/kg IV), and ampicillin (20-30 mg/kg IV) were administered preoperatively, while local analgesia was provided by intratesticular lidocaine (2 mg/kg total dose). General anesthesia was induced with alfaxalone (2–5 mg/kg IV) and maintained with isoflurane in oxygen. Postoperative care was carried out in the inpatient ward of Prof. Soeparwi Animal Hospital, with medications including amoxicillin + clavulanic acid (15 mg/kg, PO, q12h) for 5 days and topical therapy with *cDPSC-Exos* gel or Bioplacenton® once daily for 5 days. Postoperative pain was monitored daily, with tramadol (2-4 mg/kg, SC, q12h) administered for 3 days postoperatively.

Wound healing was evaluated using hematological profiles, macroscopic wound assessment, and tissue perfusion analysis with laser speckle contrast imaging (LSCI). Wound photographs were coded by an independent investigator, and all outcome assessments were performed in a blinded manner without knowledge of treatment allocation.

### cDPSC-Exos gel preparation 

The base gel used in this study contained 2% hydroxypropyl methylcellulose (HPMC) gelling agent, distilled water, 2% methyl paraben, 0.0018% propyl paraben, and 15% propylene glycol [46]. The isolated *cDPSC-Exos* were added to the base gel at a concentration of 30%, and conformed to pharmaceutical testing standards, including assessments of organoleptic properties, spreadability, adhesiveness, viscosity, and pH.

### Hematology profile 

Hematological profiles were measured preoperatively and on the fifth postoperative day as systemic parameters for inflammation and wound-healing. The parameters observed included white blood cells (WBC), leukocytes (LEU), neutrophils (NEU), eosinophils (EOS), monocytes (MON), and platelets (PLT).

### Wound-healing assessment 

The wound healing process was evaluated using a scoring system adapted from the postoperative suture-healing evolution scale [47]. Considering the specific characteristics of castration wounds in cats, the scoring system includes four parameters: (1) redness, (2) swelling, (3) pain, and (4) wound closure. Each parameter is rated on a scale of 1 to 5, with higher scores indicating better wound-healing outcomes, as shown in Table 2. Assessments were conducted immediately after surgery, and subsequently on the third and fifth days post-operation.

**Table 2:** Macroscopic wound-healing scoring.

**Parameter**	**1**	**2**	**3**	**4**	**5**
Redness (Erythema)	Mild to no redness	Mild redness	Moderate redness, <50% of wound area	Clear redness, >50% of wound area	Severe and extensive redness
Swelling (Edema)	No swelling	Mild swelling	Moderate swelling, <50% of wound area	Marked swelling, >50% of wound area	Severe swelling, tense tissue
Pain	No pain response	Mild pain response	Mild–moderate pain response	Moderate pain response	Severe pain response upon palpation
Wound closure	Open wound <10%	Open wound <25%	Open wound <50%	Open wound >50%	Open wound consistent with incision length

## LSCI

LSCI is a non-invasive technique used to assess tissue perfusion by detecting changes in laser speckle patterns generated by blood flow, thereby enabling near-real-time visualization of microcirculation [48, 49]. Digital imaging data from the LSCI-ZW Laser Speckle Contrast Imaging System (Shenzhen RWD Life Science Co., Ltd., China) were recorded on the 5th day after castration. Cats were administered gabapentin (5 mg/kg) to facilitate handling during LSCI recording. Images were acquired at a working distance of approximately 20-30 cm using a camera gain of 200, an exposure time of 20 ms, a frame rate of 1 fps, and a filter constant of 15 s. Perfusion analysis was conducted by defining circular regions of interest (ROIs) over the wound area and the surrounding tissue (1-2 cm from the wound) for comparison [49].

### Statistical analysis 

Qualitative data were analyzed descriptively, while quantitative data were analyzed statistically using IBM SPSS Statistics for Mac, version 29.0 (IBM Corp., Armonk, NY, USA). Wound scores were analyzed using the nonparametric Mann-Whitney test, while hematology and perfusion rate data were analyzed using a t-test, as the normality of the data was confirmed using the Shapiro-Wilk test. Statistical significance was set at p < 0.05. Graphs and plots were generated using GraphPad Prism (version 10.6.1), and illustrations were created using BioRender (biorender.com). 

## RESULTS

### cDPSCs revealed MSC properties 

**Figure 2:**
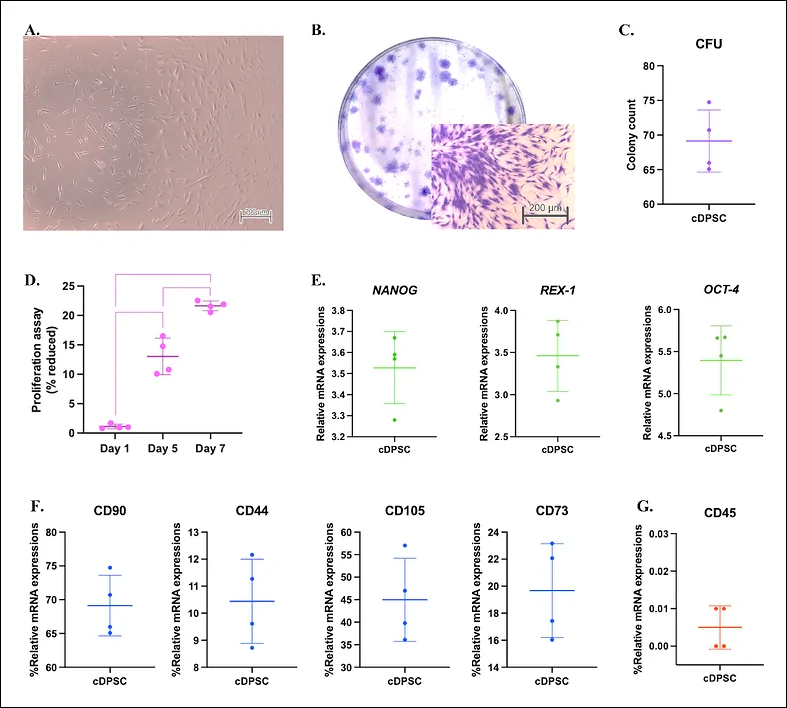
Characterization of *cDPSCs*. (A) Representative morphology of passage 3 *cDPSCs* showing spindle-like shape (100×; scale bar = 200 μm). (B-C) CFU assay showing 66.25 ± 4.11 colonies with 13.25 ± 0.82% clonogenic efficiency. (D) Cell proliferation assay performed on days 1, 5, and 7, showing a significant increase (p < 0.05). (E) Relative mRNA expression of stemness markers *NANOG*, *REX-1*, and *OCT-4*. (F) Relative mRNA expression of MSC markers *CD90*, *CD44*, *CD105*, and *CD73*. (G) Relative mRNA expression of the hematopoietic marker *CD45*.

The *cDPSCs* were successfully isolated from canine dental pulp tissue using the tissue explantation technique and characterized at passage 3, which revealed spindle-like morphology (Figure 2A), adherence to the culture dish, and showed proliferation capacity confirmed by a significant increase of proliferation rate on the 5th and 7th day (p < 0.05) (Figure 2D). The colony-forming unit (CFU) result showed 66.25 ± 4.11 colonies and 13.25 ± 0.82% clonogenic efficiency (Figures 2B and C). The self-renewal and maintenance of the non-differentiation state of isolated *cDPSCs* were confirmed by the mRNA expression of *NANOG* (3.53 ± 0.17), *OCT-4* (5.40 ± 0.42), and *REX-1* (3.46 ± 0.42) (Figure 2E). The RT-qPCR analysis indicated positive expression of MSC surface markers *CD90* (55.65 ± 21.97%), *CD44* (64.67 ± 18.57%), *CD105* (36.92 ± 8.25%), and *CD73* (34.85 ± 10.09%) (Figure 2F), and lack of hematopoietic cell surface marker *CD45* expression (0.02 ± 0.03%) (Figure 2G).

**Figure 3:**
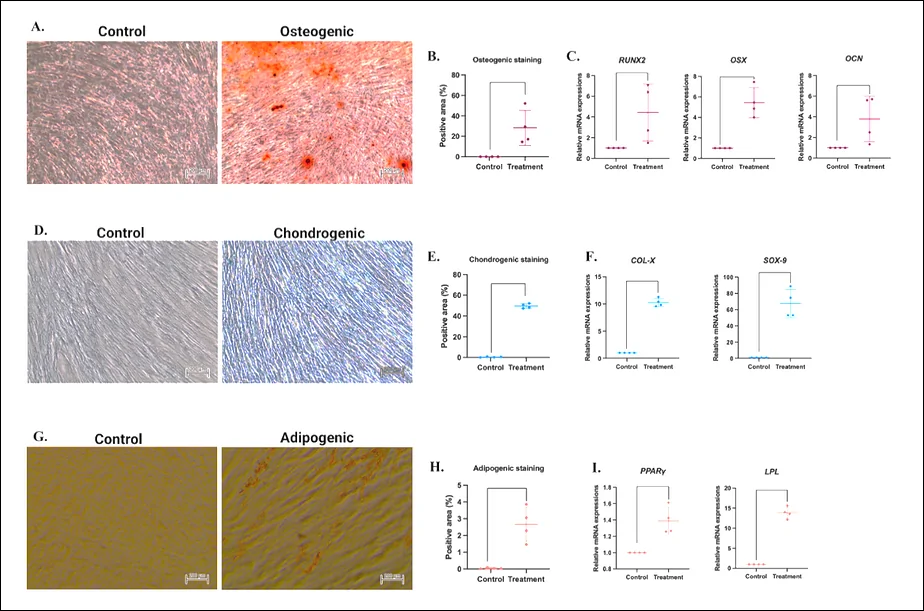
Multilineage differentiation of *cDPSCs*. (A) Osteogenic differentiation assessed by Alizarin Red staining (40×; scale bar = 200 μm), (B) quantification of Alizarin Red-positive area showing a significant increase after osteogenic induction (p < 0.05), and (C) relative mRNA expression of osteogenic markers *RUNX-2*, *OSX*, and *OCN*. (D) Chondrogenic differentiation assessed by Alcian Blue staining (40×; scale bar = 200 μm), (E) quantification of Alcian Blue-positive area showing a significant increase after chondrogenic induction (p < 0.05), and (F) relative mRNA expression of chondrogenic markers *COL-X* and *SOX-9*. (G) Adipogenic differentiation assessed by Oil Red O staining (40×; scale bar = 200 μm), (H) quantification of Oil Red O-positive area showing a significant increase after adipogenic induction (p < 0.05), and (I) relative mRNA expression of adipogenic markers *PPAR-γ* and *LPL*. Data are presented as mean ± SD (n=4). Significant differences between control and induced groups are indicated by brackets (p < 0.05).

The multipotency of *cDPSCs* in the present study was confirmed both genetically through RT-qPCR and phenotypically through multilineage staining (Figure 3). Osteogenic differentiation demonstrated by the significant upregulation (p < 0.05) of osteogenic differentiation markers *RUNX-2* (4.43 ± 2.7), *OSX* (5.45 ± 1.51) and *OCN* (3.79 ± 2.23), further confirmed by Alizarin Red staining showing mineralized matrix formation in osteogenically induced cells, with a significantly greater positive staining area in induced cells (28.29 ± 14.86%) compared with controls (0.02 ± 0.01%), corresponding to a 1370.49-fold increase (p < 0.05) (Figures 3A and B). The significant upregulation (p < 0.05) of *SOX-9* (67.34 ± 17.18) and *COL-X* (10.29 ± 0.74), supported by the Alcian Blue staining demonstrated GAG matrix synthesis in chondrogenic induced cells, with a significant increase of positive staining area from 0.36 ± 0.28% in controls to 49.59 ± 1.94% in induced cells, representing a 136.82-fold increase (p < 0.05) represent the chondrogenic differentiation potential of *cDPSCs* (Figures 3D-E). The adipogenic differentiation potential was confirmed by the significant upregulation (p < 0.05) of *PPAR-γ* (1.39 ± 0.17) and *LPL* (13.83 ± 1.49), further confirmed by the Oil Red O staining demonstrated intracellular lipid droplet accumulation in adipogenic induced cells, with a significant increase in positive staining area from 0.05 ± 0.03% in controls to 2.67 ± 0.89% in induced cells, corresponding to a 59.18-fold increase (p < 0.05) (Figures 3G–I). 

### cDPSC-Exos revealed exosome-like characteristics 

**Figure 4:**
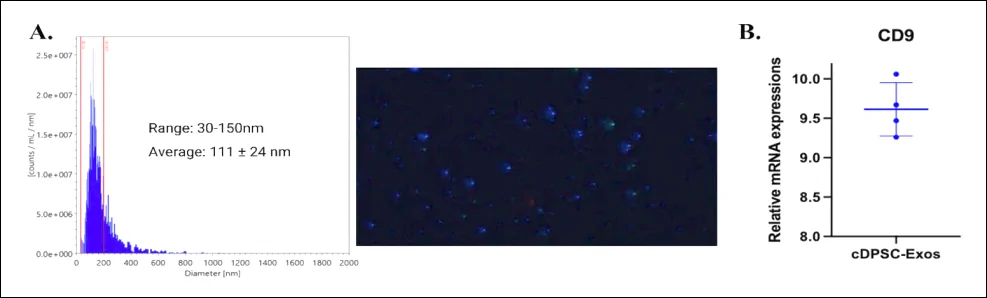
Characterization of *cDPSC*-derived exosomes (*cDPSC-Exos*). (A) Nanoparticle tracking analysis (NTA) showing the particle size distribution of isolated exosomes, with diameters ranging from 30 to 150 nm and an average diameter of 111 ± 24 nm. (B) Relative mRNA expression of the exosome marker *CD9* normalized to *GAPDH*. Data are presented as mean ± SD (n=4).

The *cDPSC-Exos* were successfully isolated using a gradual centrifugation technique combined with a precipitation-based isolation reagent. Particle size distribution predominantly within the 30-150 nm range, with an average particle size of 111 ± 24 nm and a concentration of 1.1 × 10⁹ particles/mL (Figure 4A). *CD9* transcript expression was detected in the isolated fraction (Figure 4B), providing molecular evidence supporting the presence of exosome-like components.

### cDPSC-Exos proteomic profile 

**Figure 5:**
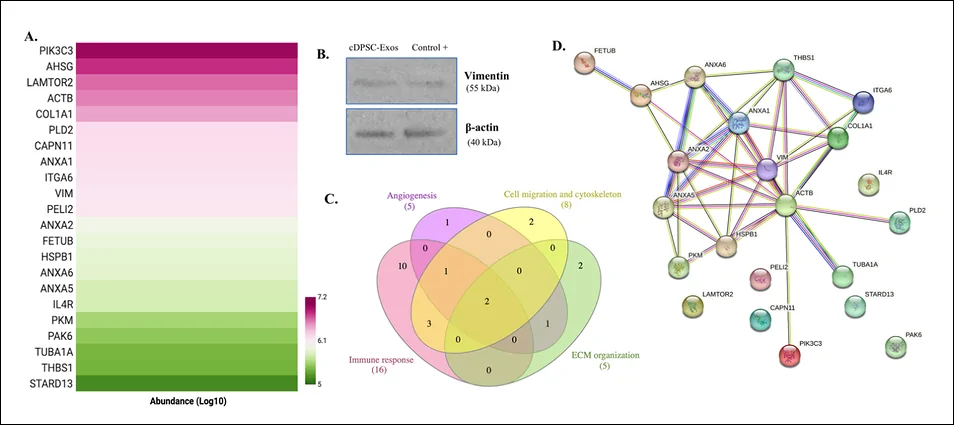
The first proteomic analysis of canine dental pulp stem cell-derived exosome (*cDPSC-Exos*). (A) Heatmap showing the relative abundance of 22 wound healing-related proteins in *cDPSC-Exos*, presented as log10 values and ranked by abundance. (B) Western blot analysis confirming vimentin (55 kDa) expression in *cDPSC-Exos*. (C) Venn diagram showing the distribution and overlap of proteins associated with angiogenesis, immune response, cell migration and cytoskeletal regulation, and extracellular matrix (ECM) organization. (D) Protein-protein interaction network generated using the STRING database for wound-healing-related proteins identified in *cDPSC-Exos*.

This is the first *cDPSC-Exos* proteomic analysis, which identifies 312 unique proteins from *Canis lupus familiaris,* including 22 involved in wound-healing-related biological processes (Figure 5). The wound-healing-related proteins of *cDPSC-Exos* were ranked from the highest abundance as follows: phosphatidylinositol 3-kinase catalytic subunit type 3 (PIK3C3), alpha-2-HS-glycoprotein (AHSG), late endosomal/lysosomal adaptor MAPK and mTOR activator 2 (LAMTOR2), actin beta (ACTB), collagen type I alpha 1 (COL1A1), phospholipase D2 (PLD2), calpain 11 (CAPN11), annexin A1 (ANXA1), integrin alpha-6 (ITGA6), vimentin (VIM), pellino E3 ubiquitin protein ligase 2 (PELI2), annexin A2 (ANXA2), fetuin-B (FETUB), heat shock protein family B member 1 (HSPB1), annexin A6 (ANXA6), annexin A5 (ANXA5), interleukin-4 receptor (IL4R), pyruvate kinase M (PKM), p21-activated kinase 6 (PAK6), tubulin alpha-1A (TUBA1A), thrombospondin-1 (THBS1), and StAR-related lipid transfer domain protein 13 (STARD13) (Figure 5A).

According to the functional analysis, these proteins were distributed across all phases of wound-healing, including immune response-related pathways in the hemostasis and inflammation phases; as well as angiogenesis, cell migration, and ECM organization during the proliferation and remodeling phases. Certain proteins were involved in multiple biological processes associated with wound-healing, as illustrated in the Venn diagram (Figure 5C). Additionally, PPI analysis (Figure 5D) revealed a complex interaction network among the wound healing-related proteins of *cDPSC-Exos*. ACTB and VIM showed the highest interactions, followed by THBS1, which showed specific interactions with members of the annexin family. These interactions indicate the involvement of *cDPSC-Exos*, particularly proteins implicated in the proliferation and remodeling phases, as evidenced by the association of COL1A1 with several cytoskeletal and ECM-related proteins. Proteins with high connectivity, including ACTB, VIM, COL1A1, and THBS1, as well as the most abundant protein PIK3C3, were involved in nearly all phases of wound-healing.

The western blot analysis revealed positive expression of vimentin (VIM) and β-actin (ACTB) in the *cDPSC-Exos* (Figure 5B). These findings were consistent with the HRMS proteomic results, which also identified ACTB and VIM among the wound-healing-related proteins. The aligned western blot and proteomic analyses support the validity of the HRMS data and suggest that the other proteins identified in the proteomic profile are also present in the *cDPSC-Exos*.

### cDPSC-Exos gel formulation

The topical gel was formulated with 3.3 × 10⁸ particles of *cDPSC-Exos* per gram of HPMC base gel to achieve a 30% gel concentration. Organoleptic evaluation showed that the *cDPSC-Exos* gel was clear, transparent, homogeneous, and odorless. Furthermore, pharmaceutical evaluation demonstrated its safety and effectiveness in delivering *cDPSC-Exos*, with a gel pH of 5, spreadability of 7.05 ± 0.24 cm, residence time of 5.32 ± 0.39 minutes, and viscosity of 5,782.75 ± 29.44 cP. The formulation was stored at 4°C prior to use, and its physical appearance was monitored through organoleptic evaluation, including clarity, homogeneity, color, and odor. The favorable pharmaceutical characteristics of the HPMC-based gel suggest it may serve as a promising delivery system for the topical administration of cDPSC-Exos in experimental wound-healing studies and clinical applications.

### Wound macroscopic evaluation 

**Figure 6:**
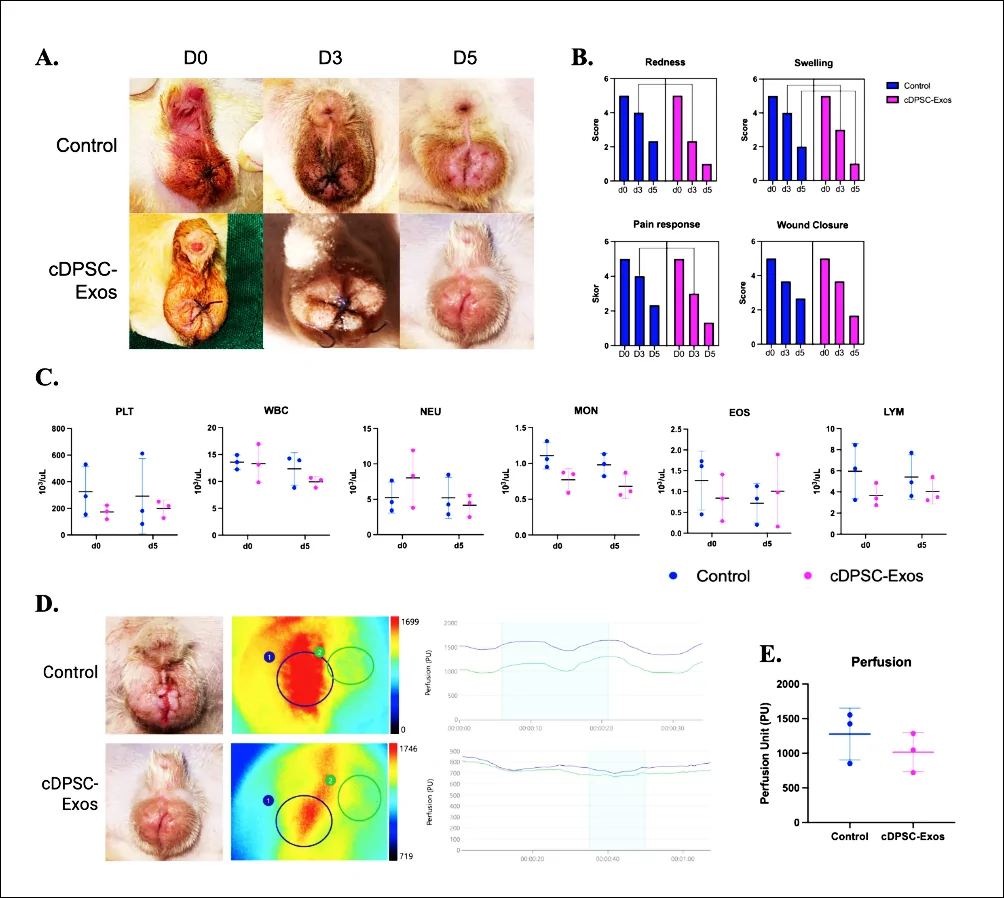
Macroscopic evaluation of post-castration wound-healing. (A) Representative wound images from the control and *cDPSC-Exos* groups at day 0 (D0), day 3 (D3), and day 5 (D5). (B) Macroscopic wound-healing scores for redness, swelling, pain response, and wound closure at each observation time point. Data are presented as mean ± SD (n = 3 cats/group), with connecting lines indicating significant differences (p < 0.05). (C) Hematological parameters including platelet (PLT), white blood cell (WBC), neutrophils (NEU), eosinophils (EOS), monocytes (MON), and lymphocytes (LYM) were measured before surgery (D0) and on day 5 (D5) post-surgery in the control and *cDPSC-Exos* groups. Data are presented as individual values with mean ± SD (n = 3 cats/group); no significant differences between groups (p > 0.05). (D) LSCI perfusion heatmaps of surgical wounds in the control and *cDPSC-Exos* groups. (E) Quantitative perfusion values expressed as perfusion units (PU). Data are presented as mean ± SD (n = 3 cats/group), no significant differences between groups (p > 0.05).

Topical application of *cDPSC-Exos* gel in this study supported post-castration wound-healing in cats without any clinical complications or adverse events, including prolonged inflammation, wound dehiscence, behavioral changes, or signs of infection. Macroscopic observation of wound-healing on days 0, 3, and 5 showed progressive improvement in both groups (Figure 6A). Macroscopic wound-healing scores between the *cDPSC-Exos* group and the positive control group showed significant differences (p < 0.05) in the redness and pain response on day 3, as well as in the swelling on days 3 and 5 (Figure 6B), while the wound closure parameters did not show significant differences (p > 0.05). However, the final wound closure score in the *cDPSC-Exos* group was lower than that in the control group, indicating improved tissue repair.

### Perfusion rate 

The LSCI results on the 5th day showed a lower perfusion rate (Speckle flow index; SFI) in the *cDPSC-Exos* group (1033.42 ± 274.61) compared to the control group (1213.41 ± 313.22), though no significant difference was observed, which may be attributed to the limited sample size (Figures 6D and 6E).

### Blood profile 

There were no significant differences in the WBC and PLT profiles between the *cDPSC-Exos* and control groups, as local inflammation rarely causes systemic alterations. Nevertheless, differences in the WBC and PLT dynamics were observed descriptively (Figure 6C). Compared to the pre-operative hematology result, a greater decrease in WBC, NEU, and MON counts, along with increased LYM and EOS counts, was observed in the *cDPSC-Exos* group on the 5th day. In contrast, the control group showed increases in WBC, NEU, and MON, while LYM and EOS decreased. A slight increase in PLT counts was observed in the cDPSC-Exos group, while the control group showed decreased PLT counts.

## DISCUSSION

### Characterization of cDPSCs 

*cDPSCs* in the present study were isolated using the tissue explantation method, exhibited clonogenic capacity comparable to hDPSCs, which ranges between 9-12% [50], and demonstrated a proliferation capacity with a growth pattern similar to hDPSCs, which exit the adaptive phase and enter the exponential phase within 3-7 days of culture [51]. Compared to the enzymatic digestion method, the tissue explant method preserves the native cellular microenvironment, has a lower risk of cellular stress and damage, is more cost-effective, supports cell attachment and outgrowth, and maintains paracrine signaling mechanisms, which may be beneficial for exosome collection [52, 53]. This native microenvironment may also contribute to the maintenance of multipotency and self-renewal in *cDPSCs*, as evidenced by the expression of stemness markers, reflecting their ability to differentiate into multiple cell lineages [54]. Furthermore, the isolated *cDPSCs* exhibited MSC characteristics, as indicated by the expression of MSC surface markers *CD90*, *CD105*, *CD73*, and *CD44* and the lack expression of the hematopoietic cell surface marker *CD45*. Although these surface markers are defined by ISCT based on human MSCs, similar expression patterns have been reported in animal-derived stem cells, including those from rabbit synovial fluid [55], equine dental pulp [7], canine bone marrow [10, 33, 34, 37, 56], as well as canine dental pulp in previous studies [33, 34, 56]. Although MSC characterization is conventionally performed using flow cytometry according to ISCT criteria, RT-qPCR was employed in the present study due to methodological limitations. Previous studies have reported consistent expression patterns of *CD90*, *CD105*, *CD73*, *CD44*, and *CD45* between RT-qPCR and flow cytometric analyses [57, 58], indicating the present findings sufficiently represent the MSC characteristics of the isolated *cDPSCs*.

The trilineage differentiation potential, including chondrogenesis, osteogenesis, and adipogenesis, serves as a functional hallmark of MSCs, as defined in the ISCT minimal requirements [59]. The isolated *cDPSCs* demons-trated trilineage differentiation potential, as assessed through phenotypic and transcriptional approaches. This differentiation capacity has also been reported in previous studies of canine-derived stem cells, including those from bone marrow, adipose tissue, and dental pulp; however, the extent of differentiation varies by tissue source [33, 34, 37, 60]. In the present study, osteogenic differentiation was the most pronounced among the three lineages, consistent with previous reports demonstrating that *cDPSCs* possess greater osteogenic potential than canine bone marrow MSCs (cBMSCs) [37], as well as a study on hDPSCs reporting enhanced osteogenic and chondrogenic capacities relative to other MSC populations [61]. This indicates that *cDPSCs'* potential is not limited to soft tissue but also to bone tissue engineering.

### Characterization and proteomic profile of cDPSC-Exos 

The successful isolation of *cDPSC*-derived exosomes in the present study demonstrates a stable and measurable yield using gradual centrifugation combined with exosome isolation reagent, as reflected by the *cDPSC-Exos* concentration within the common range of exosome isolation, 10^9^-10^10^ particle/mL [50, 62, 63]. The positive expression of *CD9* (Figure 2B) serves as molecular evidence to support exosome biogenesis from *cDPSCs*; in line with previous MSC-Exos studies in humans and animals [64–67]. However, this characterization remains preliminary, as the MISEV 2023 guidelines recommend the assessment of canonical EV markers at the protein level (*CD9*, *CD63*, *CD81*, TSG101, and Alix) along with additional characterization approaches such as ultrastructural imaging and evaluation of negative markers.

**Figure 7:**
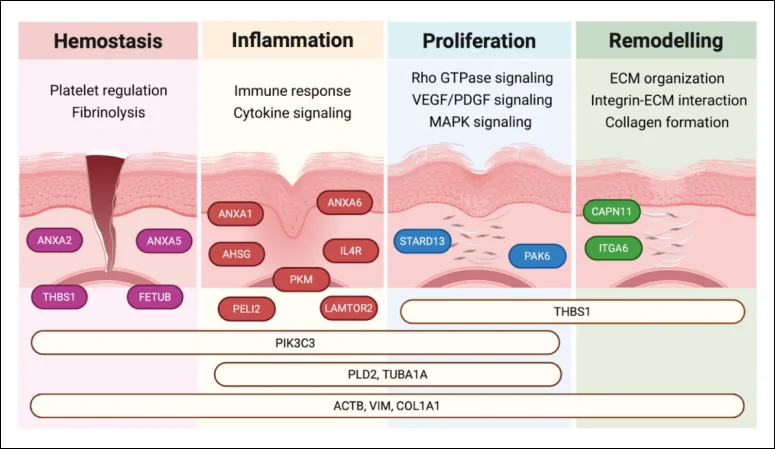
Schematic illustration of wound-healing-related proteins identified in the first *cDPSC-Exos* proteomic analysis. Proteins are grouped according to their involvement in hemostasis, inflammation, proliferation, and remodeling phases. Several proteins (PIK3C3, PLD2, VIM, TUBA1A, ACTB, COL1A1, and THBS1) are distributed across multiple phases, indicating coordinated roles in tissue repair.

This study provides the first proteomic profiling of *cDPSC-Exos*, which revealed wound healing-related proteins. These proteins were distributed across all phases of tissue repair, reflecting a coordinated and complex mechanism as illustrated in Figure 7. Notably, several proteins identified in the present study, including COL1A1, ANXA, VIM, THBS1, ITGA, and ACTB, have also been reported in proteomic studies of MSC-derived exosomes from dental pulp and various tissue sources [8, 37, 44, 68–70]. This finding suggests that *cDPSC-Exos* share molecular components commonly associated with the regenerative and wound-healing properties of MSC-derived exosomes.

Based on the functional analysis, the predominant proteins are involved in regulating the immune response during the hemostasis and inflammation phases, suggesting that *cDPSC-Exos* may primarily exert their therapeutic effects during the early stages of wound-healing, which are critical for determining subsequent tissue repair outcomes [71, 72]. Additionally, PPI analysis revealed an integrated interaction network among wound-healing-related proteins, with highly connected proteins such as ACTB and VIM highlighting the central role of cytoskeletal organization and cell motility in the biological activity of *cDPSC-Exos*. Consistent with the proteomic results, western blot analysis confirmed the expression of ACTB and VIM (Figure 3B), supporting the validity of other identified proteins. Both proteins are associated with cell migration, structural stability, and extracellular matrix (ECM) remodeling, which are essential during the proliferation and remodeling phases of wound-healing [73, 74]. During wound-healing, VIM is involved in fibroblast migration, proliferation, and differentiation, as well as in cytoskeletal remodeling and extracellular matrix organization, to promote tissue regeneration [75, 76]. ACTB is involved in wound-healing through cytoskeletal remodeling, which drives cell migration, epithelialization, and wound contraction, particularly via actin cable formation at the wound edge, thereby supporting wound closure [77]. On the other hand, VIM and ACTB revealed the biological integrity and purity of exosomes, as both proteins are associated with cytoskeleton-dependent EV biogenesis. Previous studies have demonstrated that VIM and ACTB are involved in exosome biogenesis by regulating vesicle transport, maintaining endosomal positioning, and facilitating exosome release through their interaction in vesicular transport; thus, filament assembly and disassembly influence their secretion and involvement in EV pathways [75, 78].

Based on the PPI network, THBS1 interactions with annexin family members also support the regulation of angiogenesis and membrane repair mechanisms, thereby strengthening the role of *cDPSC-Exos* in tissue regeneration [79, 80]. The high abundance of PIK3C3 may reflect its dual roles in exosome biogenesis and wound-healing regulation, as it is involved in autophagy and endocytosis, which are essential for vesicle trafficking and cellular homeostasis [81]. Autophagy mediated by PIK3C3 has been implicated in debris clearance during hemostasis, inflammasome inhibition, and macrophage polarization during inflammation, as well as cell migration, angiogenesis, and ECM remodeling during the late phases of wound repair [81–83]. The integrated proteomic analysis suggests that *cDPSC-Exos* contain proteins involved not only in exosome biogenesis but also in the comprehensive wound-healing mechanisms, supporting the therapeutic potential of *cDPSC-Exos* in promoting tissue repair.

### Xenogeneic efficacy in post-castration wound-healing 

The identified proteins may mechanistically contribute to the accelerated resolution of inflammation and wound closure observed in the present study by coordinately regulating immune responses, angiogenesis, cell migration, and extracellular matrix remodeling throughout the wound-healing process. Wound-healing involves numerous components, including blood cells, extracellular matrix, parenchymal cells, and soluble mediators, that restore tissue integrity and function through four main phases: hemostasis, inflammation, proliferation, and remodeling [47, 84]. The inflammation phase is characterized by redness, as vasodilatation increases blood flow to the wound site; swelling/edema, as increased vascular permeability allows plasma to leak into the tissue; and pain, caused by the release of mediators such as prostaglandins, cytokines, and chemokines [85]. The lower redness, swelling, and pain scores in the *cDPSC-Exos* group on day 3 indicate a faster inflammatory response than in the control group. The wound closure score represents the late stages of wound-healing, including proliferation and remodeling, characterized by the regeneration and repair of damaged skin tissue, which are relatively faster in the *cDPSC-Exos* group [47, 84]. The LSCI results were consistent with the macroscopic findings, as a lower perfusion unit indicates *that the cDPSC-Exos* group entered the remodeling phase on day 5. Active angiogenesis occurs in the early stage of the wound-healing process, resulting in a higher SFI value as assessed by LSCI, which gradually decreases during the remodeling phase, approaching that of normal skin tissue [86]. Angiogenesis forms new blood vessels from the existing vasculature, thereby maintaining oxygen and nutrient supply to damaged skin tissue and playing an important role in tissue regeneration and wound-healing [87].

The blood profile was consistent with the macroscopic and LSCI results, indicating accelerated wound-healing in the *cDPSC-Exos* group. Platelets play an important role in hemostasis, and the slight increase in platelet counts in the *cDPSC-Exos* group may indicate the end of hemostasis as platelet counts return to normal [84, 85]. During the inflammatory phase, neutrophils and macrophages infiltrate the wounded skin to fight microorganisms, clear cellular debris, and release inflammatory mediators; therefore, a decrease in neutrophil and monocyte counts indicates resolution of the inflammatory phase [84]. The neutrophil and monocyte trends suggest that the inflammatory phase in the *cDPSC-Exos* group occurred over a relatively shorter period than in the control group, which may be associated with the abundance of immune-related proteins in *cDPSC-Exos*. Increased lymphocytes and eosinophils may indicate the onset of the late stage of wound-healing, as they play important roles in the proliferative and remodeling phases. EOS interact with the coagulation and fibrinolytic systems, promoting ECM modification and epithelial repair by releasing growth factors and proteases, while lymphocytes regulate tissue regeneration and epithelial barrier restoration through cytokine-mediated immune modulation [88, 89]. These findings were comparable between groups and suggest a trend toward improved wound-healing in the *cDPSC-Exos* group compared with the control group, although no statistically significant difference was observed. The improvement in wound-healing is consistent with previous studies of MSC-derived exosomes and stem cell-based therapies in companion animals, which have similarly reported enhanced tissue repair [5, 15, 17, 90].

### Safety, limitations, and future directions 

The absence of treatment-related adverse clinical reactions following xenogeneic *cDPSC-Exos* administration provides preliminary evidence supporting the feasibility of cross-species cell-free regenerative approaches. Similar findings have been reported in previous studies demonstrating the low immunogenicity of MSC-derived exosomes across species barriers, including hu-AdMSCs secretome in feline chronic gingivostomatitis therapy, human cell exosomes in canine atopic dermatitis treatment, and numerous human MSC-Exos studies revealed acceleration in animal model wound-healing [6, 14, 15, 18, 19]. Although no evidence of xenogeneic adverse reactions was observed, the immunological safety of *cDPSC-Exos* in this study warrants further investigation. The comprehensive approach adopted in the present study, integrating proteomic profiling, hematological assessment, tissue perfusion analysis, and clinical wound evaluation, provides complementary information to understand the biological effects of *cDPSC-Exos*.

This pilot study has several limitations, particularly that the wound healing evaluation was based solely on clinical assessments, without histological or molecular assessments to characterize tissue regeneration and the cellular mechanisms of wound-healing. In addition, the relatively small sample size and limited wound size may limit the interpretation of the findings. Further studies including histopathology, immunohistochemistry, and comprehensive immunological analyses of wound tissue are needed to confirm the safety of *cDPSC-Exos* xenogeneic application. Despite these limitations, the present findings provide preliminary evidence of the potential of cDPSC-Exos in feline wound-healing. As a cell-free therapeutic approach, *cDPSC-Exos* may have translational potential in veterinary medicine, although further standardization, safety evaluation, and regulatory considerations are needed before broader clinical application.

## CONCLUSION

*cDPSC-Exos* demonstrated promising therapeutic potential for postoperative wound-healing in cats, as indicated by accelerated wound healing parameters and the presence of angiogenic, anti-inflammatory, and immunomodulatory proteins identified by proteomic analysis. This study represents the first report describing the isolation, characterization, proteomic profiling, and xenogeneic topical application of *cDPSC-Exos* in a feline surgical wound model, and pioneers the use of companion-animal dental pulp as a sustainable source for xenogeneic cell-free products, offering a practical pathway for clinical veterinary regenerative medicine.

Proteomic analysis identified 312 unique proteins in *cDPSC-Exos*, including 22 associated with wound healing processes across all phases (hemostasis, inflammation, proliferation, and remodeling). In the pilot *in vivo* study, topical *cDPSC-Exos* gel (3.3 × 10⁸ particles/g) significantly improved early macroscopic wound scores for redness, swelling, and pain (p < 0.05) compared with Bioplacenton®, with supportive trends in hematological parameters and tissue perfusion (LSCI), and no adverse reactions observed.

These findings support *cDPSC-Exos* as a safe, xenogeneic, cell-free regenerative therapy derived from a minimally invasive dental pulp source. This approach offers a scalable alternative to cell-based therapies for post-surgical wound management in companion animals, with potential for broader veterinary applications.

The study integrates comprehensive *in vitro* characterization, detailed proteomic profiling, and a randomized controlled pilot *in vivo* evaluation in a clinically relevant feline post-castration model, providing strong preliminary evidence of both molecular mechanisms and functional efficacy.

This pilot study has several limitations, particularly that the wound healing evaluation was based solely on clinical assessments, without histological or molecular assessments to characterize tissue regeneration and the cellular mechanisms of wound-healing. In addition, the relatively small sample size and limited wound size may limit the interpretation of the findings.

Further studies, including histopathology, immunohistochemistry, and comprehensive immunological analyses of wound tissue, are needed to confirm the safety of the application of *xenogeneic cDPSC-Exo*. Larger well-powered randomized controlled trials, extended observation periods, optimized dosing and formulation, and mechanistic investigations are required to validate efficacy and support clinical translation. Standardization, long-term safety evaluation, and regulatory considerations will also be essential before broader application.

Despite these limitations, the present findings provide preliminary evidence of *cDPSC-Exos*' potential for feline wound healing. As a cell-free therapeutic approach, *cDPSC-Exos* may have translational potential in veterinary medicine. Given the pilot nature of the study, the limited sample size, and the absence of histological and molecular wound assessments, further well-powered studies with comprehensive exosome characterization, histopathological evaluation, and mechanistic investigations are required to validate these preliminary findings and support future clinical translation of *cDPSC-Exos* as a cell-free regenerative therapy in veterinary medicine.

## DATA AVAILABILITY

The data supporting the findings of this study are available in this article and its Supplementary Materials, including the raw NTA data, quantification of multilineage differentiation staining, full-length western blot images, the proteomic dataset, detailed animal information, raw wound healing data, and the ARRIVE 2.0 checklist. Supplementary materials and additional data are available from the corresponding author upon reasonable request.

## GENERATIVE AI DECLARATION

Generative AI tools were used solely to improve language, grammar, and readability, and were not listed as authors. All scientific content, analysis, interpretation, and conclusions were developed and verified by the authors, who take full responsibility for the accuracy and integrity of the work.

## AUTHORS’ CONTRIBUTIONS

MP: Conceptualized and designed the study. YSD and MP: Conducted the animal experiments, interpreted the data, performed the statistical analysis, and drafted the manuscript. DA: Performed the castration surgeries. AH and TB: Contributed to stem cell characterization and data interpretation. CS and SN: Contributed to exosome characterization, critically reviewed, and edited the manuscript. MP, YSD, AH, TB, CS, and SN: Revised and approved the final manuscript. All authors have read and approved the final manuscript.

## References

[1] Mai Z, Chen H, Ye Y, Hu Z, Sun W, Cui L (2021). Translational and clinical applications of dental stem cell-derived exosomes. Front Genet.

[2] Mei R, Wan Z, Yang C, Shen X, Wang R, Zhang H (2024). Advances and clinical challenges of mesenchymal stem cell therapy. Front Immunol.

[3] Yuan M, Hu X, Yao L, Jiang Y, Li L (2022). Mesenchymal stem cell homing to improve therapeutic efficacy in liver disease.

[4] Han X, Liao R, Li X, Zhang C, Huo S, Qin L (2025). Mesenchymal stem cells in treating human diseases: molecular mechanisms and clinical studies.

[5] Purbantoro SD, Taephatthanasagon T, Purwaningrum M, Hirankanokchot T, Peralta S, Fiani N (2024). Trends of regenerative tissue engineering for oral and maxillofacial reconstruction in veterinary medicine. Front Vet Sci.

[6] Zhou Y, Zhang XL, Lu ST, Zhang NY, Zhang HJ, Zhang J (2022). Human adipose-derived mesenchymal stem cells-derived exosomes encapsulated in pluronic F127 hydrogel promote wound healing and regeneration. Stem Cell Res Ther.

[7] Purwaningrum M, Haryanto A, Kayanaveda Y, Sawangmake C (2025). Osteogenic differentiation potential of equine dental pulp vs.

[8] Villatoro AJ, Martín-Astorga MDC, Alcoholado C, Sánchez-Martín MDM, Becerra J (2021). Proteomic analysis of the secretome and exosomes of feline adipose-derived mesenchymal stem cells. Animals.

[9] Lin CS, Xin ZC, Dai J, Lue TF (2013). Commonly used mesenchymal stem cell markers and tracking labels: limitations and challenges. Histol Histopathol.

[10] Purwaningrum M, Jamilah NS, Purbantoro SD, Sawangmake C, Nantavisai S (2021). Comparative characteristic study from bone marrow-derived mesenchymal stem cells. J Vet Sci.

[11] Zhou Z, Zheng J, Lin D, Xu R, Chen Y, Hu X (2022). Exosomes derived from dental pulp stem cells accelerate cutaneous wound healing by enhancing angiogenesis via the Cdc42/p38 MAPK pathway.

[12] Tan F, Li X, Wang Z, Li J, Shahzad K, Zheng J (2024). Clinical applications of stem cell-derived exosomes.

[13] Pamulang YV, Oontawee S, Rodprasert W, Padeta I, Sa-Ard-lam N, Mahanonda R (2025). Potential upscaling protocol establishment and wound healing bioactivity screening of exosomes isolated from canine adipose-derived mesenchymal stem cells. Sci Rep.

[14] Zhang L, Shi C, Yan L, Zhang X, Ji X, Li L (2025). The application of exosomes from different sources loaded with natural small-molecule compounds in disease. Int J Nanomedicine.

[15] Almendros A, Nekouei O, Moores C, Jesky R, Baiker K, Tse M (2025). Case report: xenogeneic mesenchymal stem cell secretome for the treatment of feline chronic gingivostomatitis. Front Vet Sci.

[16] Yang Y, Huang Y, Yang J, Hu Z, Wu S, Yuan Q (2025). Umbilical cord mesenchymal stem cell-derived exosomes promote wound healing and skin regeneration via the regulation of inflammation and angiogenesis. Front Bioeng Biotechnol.

[17] Chae CW, Kim DH, Jo HY, Oh YJ, Lee HJ (2025). Exosomes-based applications in companion animals: diagnostics and therapeutics in dogs and cats.

[18] Kim SW, Lim KM, Cho SG, Ryu B, Kim CY, Park SY (2024). Efficacy of allogeneic and xenogeneic exosomes for the treatment of canine atopic dermatitis: a pilot study. Animals.

[19] Qiao X, Tang J, Dou L, Yang S, Sun Y, Mao H (2023). Dental pulp stem cell-derived exosomes regulate anti-inflammatory and osteogenesis in periodontal ligament stem cells and promote the repair of experimental periodontitis in rats. Int J Nanomedicine.

[20] Nakazaki M, Lankford KL, Toyoshima M, Tanaka Y, Sumida TS, Kocsis JD (2026). Continuous intravenous infusion of human mesenchymal stromal cell-derived small extracellular vesicles in spinal cord injured rat modulates extracellular matrix and has greater therapeutic efficacy than multiple single injections.

[21] Schuh CMAP, Cuenca J, Alcayaga-Miranda F, Khoury M (2019). Exosomes on the border of species and kingdom intercommunication. Transl Res.

[22] Vonk LA, Frank RM (2025). Preclinical evidence of MSC-derived exosomes.

[23] Lu X, Xu R, Dong X, Bai D, Ji W, Chen X (2025). Cell-derived exosome therapy for diabetic peripheral neuropathy: a preclinical animal studies systematic review and meta-analysis. Stem Cell Res Ther.

[24] Mou C, Xia Z, Wang X, Dai X, Wang J, Zhang C (2025). Stem cell-derived exosome treatment for acute spinal cord injury: a systematic review and meta-analysis based on preclinical evidence. Front Neurol.

[25] Wang Y, Kong Y, Du J, Qi L, Liu M, Xie S (2025). Injection of human umbilical cord mesenchymal stem cells exosomes for the treatment of knee osteoarthritis: from preclinical to clinical research. J Transl Med.

[26] Zhou L, Cai W, Zhang Y, Zhong W, He P, Ren J (2025). Therapeutic effect of mesenchymal stem cell-derived exosome therapy for periodontal regeneration: a systematic review and meta-analysis of preclinical trials. J Orthop Surg Res.

[27] Quan J, Liu Q, Li P, Yang Z, Zhang Y, Zhao F (2025). Mesenchymal stem cell exosome therapy: current research status in the treatment of neurodegenerative diseases and the possibility of reversing normal brain aging.

[28] Zhou C, Zhang B, Yang Y, Jiang Q, Li T, Gong J (2023). Stem cell-derived exosomes: emerging therapeutic opportunities for wound healing. Stem Cell Res Ther.

[29] Chutipongvivate P, Homkong P, Chanachai K (2019). Risk factors associated with post-operative wound complications in the animal birth control program, Chiang Mai Municipality, Thailand, 2017. OSIR J.

[30] Farooq A, Javid M, Murtaza S, Lashari M, Shah M, Saleem M (2023). Post-operative complications encountered by the students during canine surgery: a retrospective study. Biol Clin Sci Res J.

[31] Espinel-Rupérez J, Martín-Ríos MD, Salazar V, Baquero-Artigao MR, Ortiz-Díez G (2019). Incidence of surgical site infection in dogs undergoing soft tissue surgery: risk factors and economic impact. Vet Rec Open.

[32] Rigby BE, Malott K, Hetzel SJ, Soukup JW (2021). Incidence and risk factors for surgical site infections following oromaxillofacial oncologic surgery in dogs. Front Vet Sci.

[33] Dissanayaka WL, Zhu X, Zhang C, Jin L (2011). Characterization of dental pulp stem cells isolated from canine premolars.

[34] Utumi PH, Fracaro L, Senegaglia AC, Fragoso FYI, Miyasaki DM, Rebelatto CLK (2021). Canine dental pulp and umbilical cord-derived mesenchymal stem cells as alternative sources for cell therapy in dogs.

[35] Purbantoro SD, Osathanon T, Nantavisai S, Sawangmake C (2022). Osteogenic growth peptide enhances osteogenic differentiation of human periodontal ligament stem cells. Heliyon.

[36] Purwaningrum M (2021). The role of interleukin-6 (IL-6) on osteogenic differentiation potential of human periodontal ligament stem cells (hPDLSCs) in vitro [Internet].

[37] Nantavisai S, Pisitkun T, Osathanon T, Pavasant P, Kalpravidh C, Dhitavat S (2020). Systems biology analysis of osteogenic differentiation behavior by canine mesenchymal stem cells derived from bone marrow and dental pulp. Sci Rep.

[38] Purwaningrum M, Giachelli CM, Osathanon T, Rattanapuchpong S, Sawangmake C (2023). Dissecting specific Wnt components governing osteogenic differentiation potential by human periodontal ligament stem cells through interleukin-6. Sci Rep.

[39] Ahmad P, Siddiqui DA, Bianchi-Smak J, Farshidfar N, Estrin N, Miron RJ (2026). Effects of periodontal-specific exosomes and rhBMP2 on osteogenic behaviour and differentiation of BMSCs.

[40] Blümke A, Ijeoma E, Simon J, Wellington R, Purwaningrum M, Doulatov S (2023). Comparison of osteoclast differentiation protocols from human induced pluripotent stem cells of different tissue origins. Stem Cell Res Ther.

[41] Sholihah IA, Barlian A (2024). Anti-inflammatory potency of human Wharton’s jelly mesenchymal stem cell-derived exosomes on L2 cell line induced by lipopolysaccharides. Adv Pharm Bull.

[42] Choi EW, Lim IR, Park JH, Song J, Choi B, Kim S (2023). Exosomes derived from mesenchymal stem cells primed with disease-condition-serum improved therapeutic efficacy in a mouse rheumatoid arthritis model via enhanced TGF-β1 production. Stem Cell Res Ther.

[43] Calligaris M, Zito G, Busà R, Bulati M, Iannolo G, Gallo A (2024). Proteomic analysis and functional validation reveal distinct therapeutic capabilities related to priming of mesenchymal stromal/stem cells with IFN-γ and hypoxia: potential implications for their clinical use. Front Cell Dev Biol.

[44] Park J, Lee J, Kim YS, Oh Y (2025). Proteomic profiling of exosomes derived from endometrial stem cells and adipose-derived stem cells.

[45] Sormunen A, Koivulehto E, Alitalo K, Saksela K, Laham-Karam N, Ylä-Herttuala S (2023). Comparison of automated and traditional western blotting methods. Methods Protoc.

[46] Vlad RA, Pintea A, Pintea C, Rédai EM, Antonoaea P, Bîrsan M (2025). Hydroxypropyl methylcellulose—a key excipient in pharmaceutical drug delivery systems. Pharmaceutics.

[47] Carreira LM, Silva R, Alves J, Inácio F, Pires G, Azevedo P (2024). The use of fast-acting insulin topical solution on skin to promote surgical wound healing in cats. Animals.

[48] Linkous C, Pagan AD, Shope C, Andrews L, Snyder A, Ye T (2023). Applications of laser speckle contrast imaging technology in dermatology. JID Innov.

[49] Chen Z, Cheng Z, Chen G, Liao X, Zhao Y, Yang C (2025). A clinical research: blood perfusion of artificial dermis detected by laser speckle contrast imaging influence the therapeutic effect on wound healing. Lasers Med Sci.

[50] Alsulaimani RS, Ajlan SA, Aldahmash AM, Alnabaheen MS, Ashri NY (2016). Isolation of dental pulp stem cells from a single donor and characterization of their ability to differentiate after 2 years of cryopreservation. Saudi Med J.

[51] Qu G, Li Y, Chen L, Chen Q, Zou D, Yang C (2021). Comparison of osteogenic differentiation potential of human dental-derived stem cells isolated from dental pulp, periodontal ligament, dental follicle, and alveolar bone. Stem Cells Int.

[52] Hendijani F (2017). Explant culture: an advantageous method for isolation of mesenchymal stem cells from human tissues.

[53] Kestendjieva S, Chervenkov M, Oreshkova T, Mourdjeva M, Stoyanova E (2024). Mesenchymal stromal/stem cells isolated by explant culture method from Wharton’s jelly and subamnion possess similar biological characteristics. Appl Sci (Basel).

[54] Gabr HM, El-Kheir WA, Gabr HM, El-Kheir WA (2022). Stem cells: definition, biological types, classifications, and properties.

[55] Akgün EE, Bilici E (2025). The neurogenic differentiation of rabbit synovial fluid mesenchymal stem cells. Turk J Vet Res.

[56] Marx R, Nemec A, Kocjan A, Voga M (2026). In vitro effects of different biomaterials on canine dental pulp stem cells. Front Vet Sci.

[57] Pham H, Tonai R, Wu M, Birtolo C, Chen M (2018). CD73, CD90, CD105 and cadherin-11 RT-PCR screening for mesenchymal stem cells from cryopreserved human cord tissue. Int J Stem Cells.

[58] Bryl R, Dompe C, Jankowski M, Stefańska K, Narenji AG, Kulus J (2020). QPCR analysis of mesenchymal stem cell marker expression during the long-term culture of canine adipocyte derived stem cells. Med J Cell Biol.

[59] Dominici M, Le Blanc K, Mueller I, Slaper-Cortenbach I, Marini FC, Krause DS (2006). Minimal criteria for defining multipotent mesenchymal stromal cells. The International Society for Cellular Therapy position statement. Cytotherapy.

[60] Phyo H, Aburza A, Mellanby K, Esteves CL (2023). Characterization of canine adipose- and endometrium-derived mesenchymal stem/stromal cells and response to lipopolysaccharide. Front Vet Sci.

[61] Yasui T, Mabuchi Y, Morikawa S, Onizawa K, Akazawa C, Nakagawa T (2017). Isolation of dental pulp stem cells with high osteogenic potential. Inflamm Regen.

[62] Sung SE, Seo MS, Kang KK, Choi JH, Lee SJ, Lim JH (2022). Isolation and characterization of extracellular vesicle from mesenchymal stem cells of the epidural fat of the spine. Asian Spine J.

[63] Ahmad P, Estrin N, Farshidfar N, Zhang Y, Miron RJ (2025). Isolation methods of exosomes derived from dental stem cells. Int J Oral Sci.

[64] Huang YC, Chang CY, Huang CJ (2025). Effectiveness of exosomes from different mesenchymal stem cells in the treatment of psoriasis: a murine study and meta-analysis of experimental studies. Biomedicines.

[65] Khongkla E, Promtap K, Meerasri J, Mo-Mai P, Chankamngoen W, Sirinonthanawech N (2026). Human adipose stem cell-derived exosomes modulate the transcriptome of D-galactose-induced neuronal cells. Sci Rep.

[66] Klymiuk MC, Balz N, Elashry MI, Heimann M, Wenisch S, Arnhold S (2019). Exosomes isolation and identification from equine mesenchymal stem cells. BMC Vet Res.

[67] Furuta T, Miyaki S, Ishitobi H, Ogura T, Kato Y, Kamei N (2016). Mesenchymal stem cell-derived exosomes promote fracture healing in a mouse model. Stem Cells Transl Med.

[68] Hirakawa T, Kato T, Nakanishi Y, Urushiyama D, Miyata K, Baba T (2025). Proteomic and metabolomic analyses of adipose-derived mesenchymal stem cell exosomes and culture supernatants. Anticancer Res.

[69] Hodgson-Garms M, Moore MJ, Martino MM, Kelly K, Frith JE (2025). Proteomic profiling of iPSC and tissue-derived MSC secretomes reveal a global signature of inflammatory licensing.

[70] Li S, Zhang J, Liu X, Wang N, Sun L, Liu J (2024). Proteomic characterization of hUC-MSC extracellular vesicles and evaluation of its therapeutic potential to treat Alzheimer’s disease.

[71] Fernández-Guarino M, Hernández-Bule ML, Bacci S (2023). Cellular and molecular processes in wound healing. Biomedicines.

[72] Mamun A Al, Shao C, Geng P, Wang S, Xiao J (2024). Recent advances in molecular mechanisms of skin wound healing and its treatments.

[73] Walker JL, Bleaken BM, Romisher AR, Alnwibit AA, Menko AS (2018). In wound repair vimentin mediates the transition of mesenchymal leader cells to a myofibroblast phenotype. Mol Biol Cell.

[74] Ahangar P, Strudwick XL, Cowin AJ (2022). Wound healing from an actin cytoskeletal perspective.

[75] Yuan Z, Janmey PA, McCulloch CA (2025). Structure and function of vimentin in the generation and secretion of extracellular vimentin in response to inflammation.

[76] Parvanian S, Zha H, Su D, Xi L, Jiu Y, Chen H (2021). Exosomal vimentin from adipocyte progenitors protects fibroblasts against osmotic stress and inhibits apoptosis to enhance wound healing.

[77] Takaya K, Okabe K, Sakai S, Aramaki-Hattori N, Asou T, Kishi K (2024). Salicylate induces epithelial actin reorganization via activation of the AMP-activated protein kinase and promotes wound healing and contraction in mice.

[78] Wu J, Xie Q, Liu Y, Gao Y, Qu Z, Mo L (2021). A small vimentin-binding molecule blocks cancer exosome release and reduces cancer cell mobility.

[79] Zhang X, Zheng J, Zhang L, Zhang J, Feng L, Zhang L (2025). Transcriptomic and proteomic integrated analysis reveals molecular mechanisms of 3D bioprinted vaginal scaffolds in vaginal regeneration. Sci Rep.

[80] Gerke V, Gavins FNE, Geisow M, Grewal T, Jaiswal JK, Nylandsted J (2024). Annexins—a family of proteins with distinctive tastes for cell signaling and membrane dynamics. Nat Commun.

[81] Shen Y, Gleghorn JP (2025). Class III phosphatidylinositol-3 kinase/vacuolar protein sorting 34 in cardiovascular health and disease.

[82] Wei J, Xu F, Wei X, Zhao J, Liu J (2025). PIK3C3 influences immune cell function by modulating autophagy to exert anti-inflammatory effects in sepsis. Gene.

[83] Jin W, Ren D, Yu M, Li Y, Zhang W, Guo S (2025). Selective autophagy: a potential player in cutaneous wound healing. Adv Wound Care (New Rochelle).

[84] Feng P, Luo Y, Ke C, Qiu H, Wang W, Zhu Y (2021). Chitosan-based functional materials for skin wound repair: mechanisms and applications. Front Bioeng Biotechnol.

[85] Ji RR, Cheng J, Ji J (2023). Neuroimmune interactions in pain: mechanisms and therapeutics.

[86] Chelmu Voda C, Stefanopol IA, Gurau G, Hîncu MA, Popa GV, Mateescu OG (2025). Update on the study of angiogenesis in surgical wounds in patients with childhood obesity.

[87] Min Q, Yang L, Tian H, Tang L, Xiao Z, Shen J (2023). Immunomodulatory mechanism and potential application of dental pulp-derived stem cells in immune-mediated diseases.

[88] Jakovija A, Chtanova T (2023). Skin immunity in wound healing and cancer. Front Immunol.

[89] Coden ME, Berdnikovs S (2020). Eosinophils in wound healing and epithelial remodeling: is coagulation a missing link? J Leukoc Biol.

[90] Yilmaz Z, Varlik T, Levent Karabarut P, Ünlüişler Ş, Canikyan S (2024). Topical xenogeneic exosome therapy in a dog with toxic epidermal necrolysis. Kafkas Univ Vet Fak Derg.

